# Supplementary figures and images for: p62 sorts Lupus La and selected microRNAs into breast cancer-derived exosomes
Source: J Cell Biol. 2025 Dec 26;225(3):e202503087. doi: 10.1083/jcb.202503087 (PMC12755392; doi:10.1083/jcb.202503087)

Figure 1A

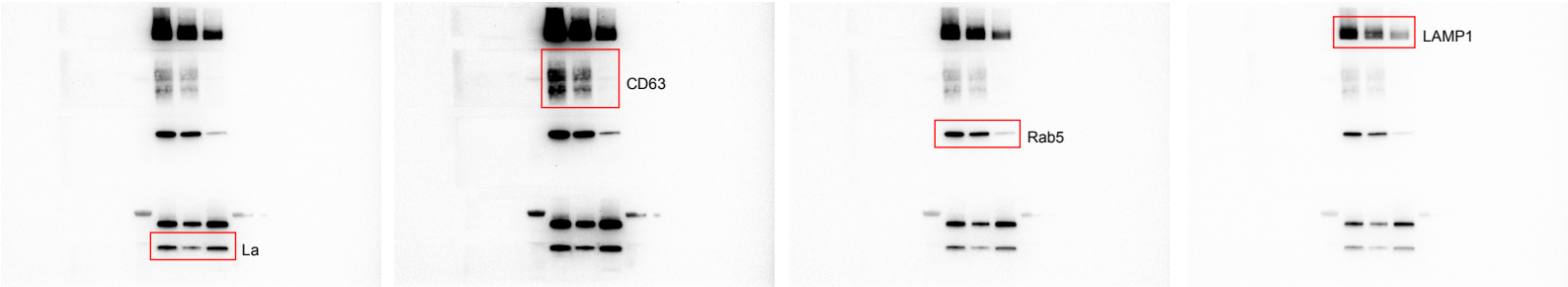

Figure 1C

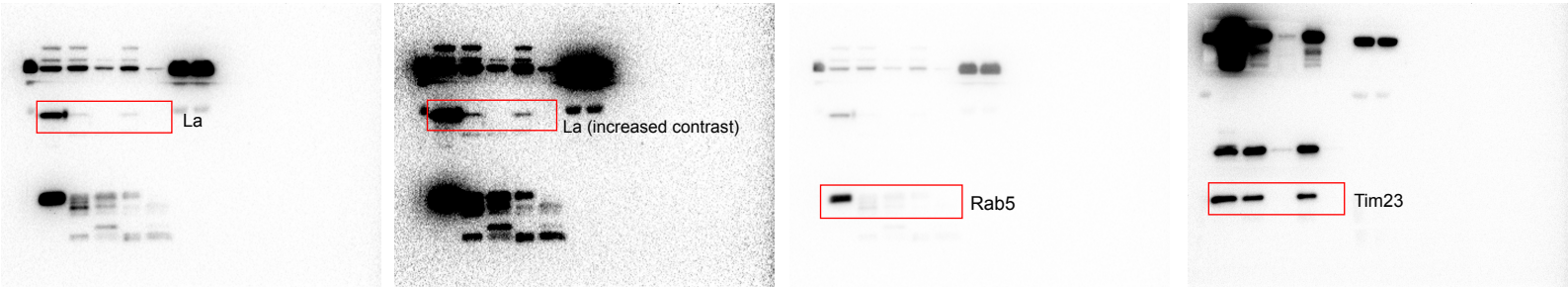

Source Data - Figure 1

Supplement: SourceData F1 — is the source file for Fig. 1. [file jcb_202503087_sourcedataf1.pdf]

Figure 3A

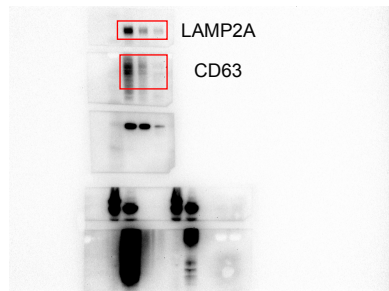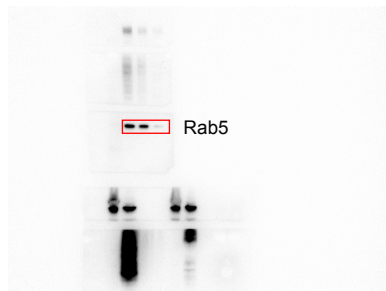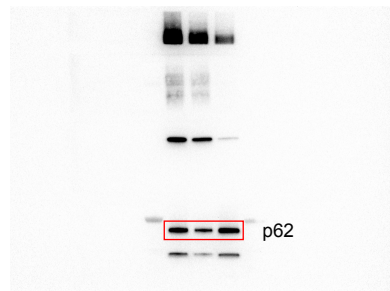

Figure 3C

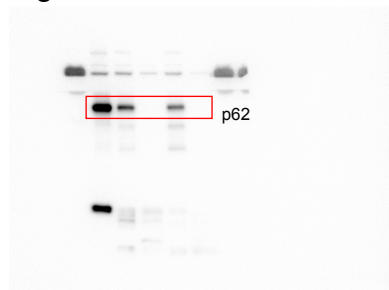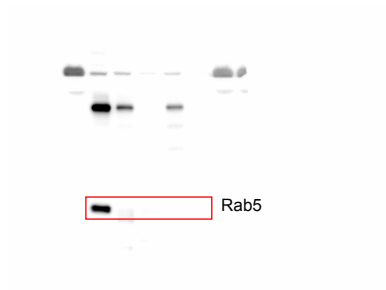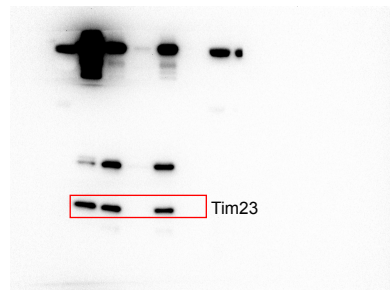

Supplement: SourceData F3 — is the source file for Fig. 3. [file jcb_202503087_sourcedataf3.pdf]

Figure 4B

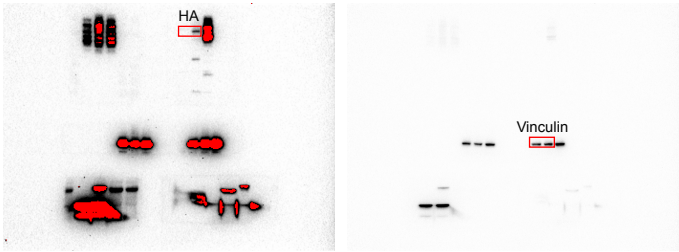

Figure 4D

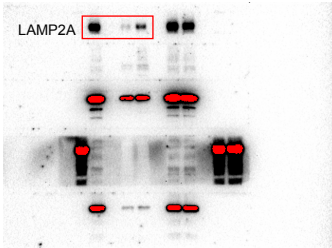

Figure 4F

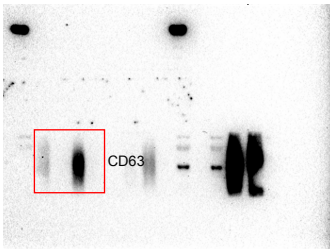

Figure 4G

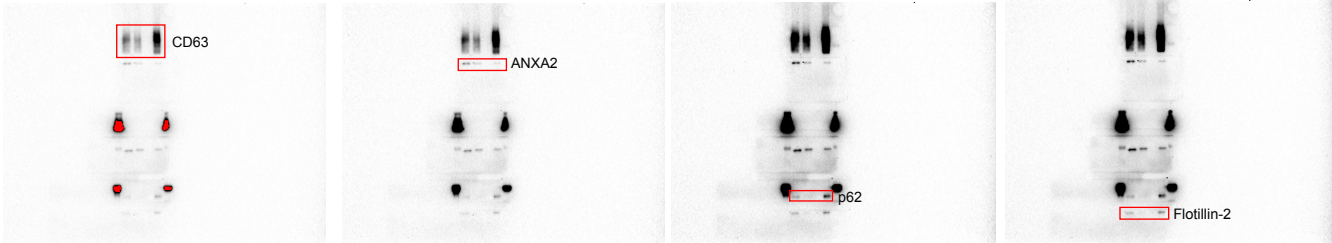

Figure 4H

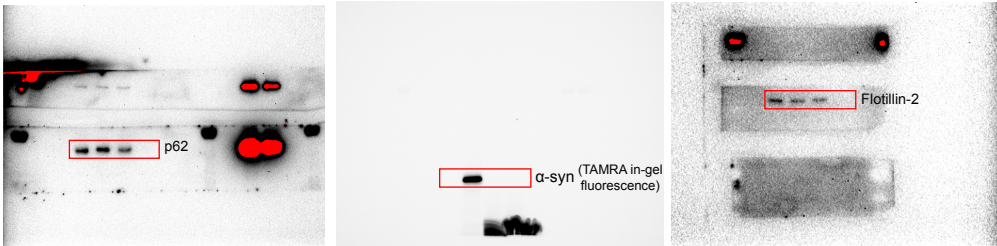

Supplement: SourceData F4 — is the source file for Fig. 4. [file jcb_202503087_sourcedataf4.pdf]

Figure 5A

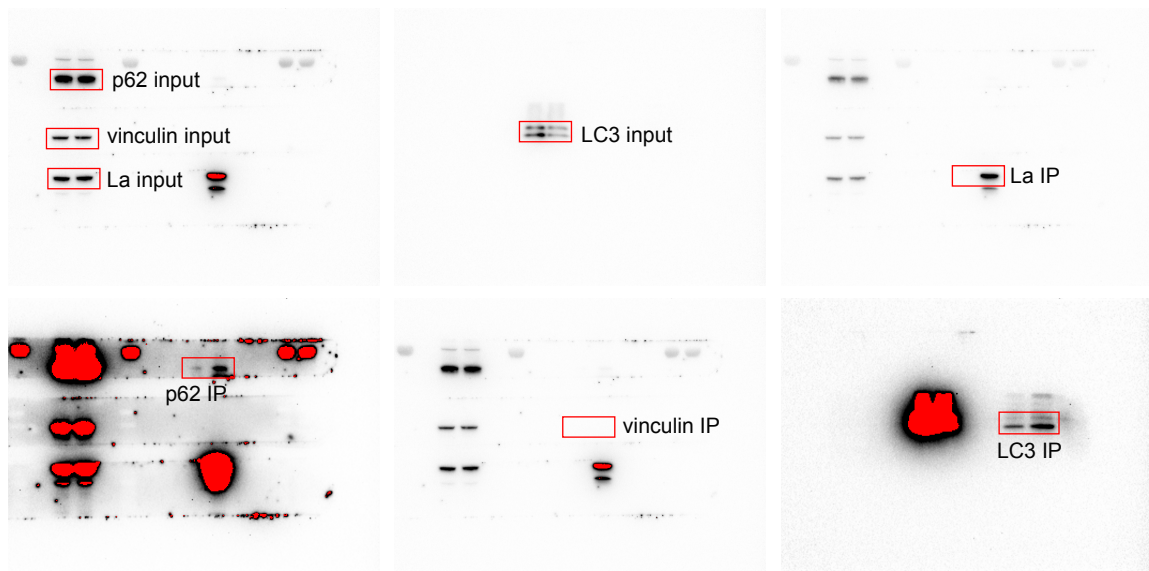

Figure 5B

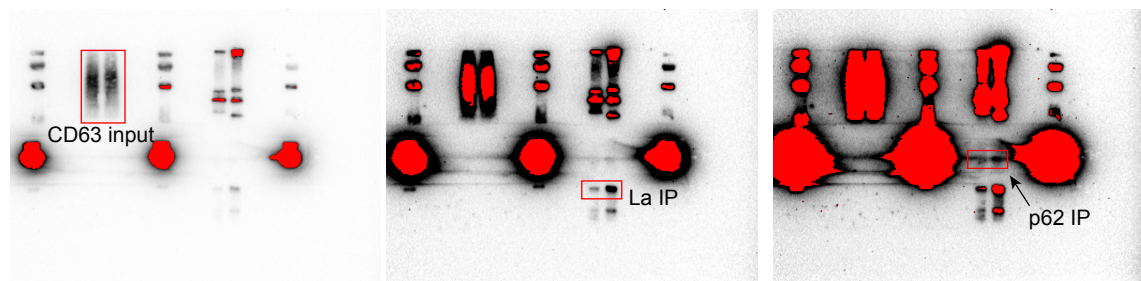

Figure 5C

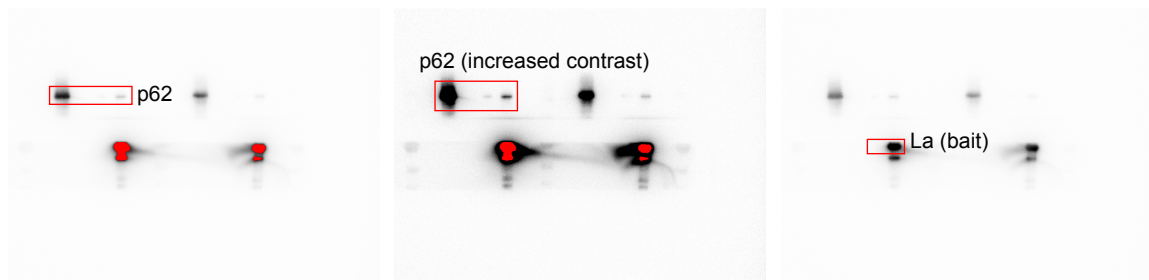

Supplement: SourceData F5 — is the source file for Fig. 5. [file jcb_202503087_sourcedataf5.pdf]

Figure 6B

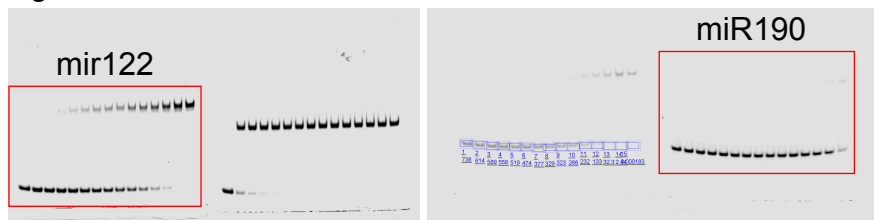

Figure 6D

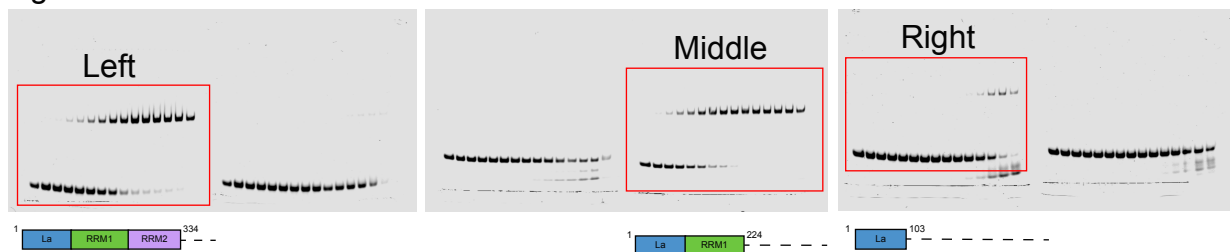

Figure 6F

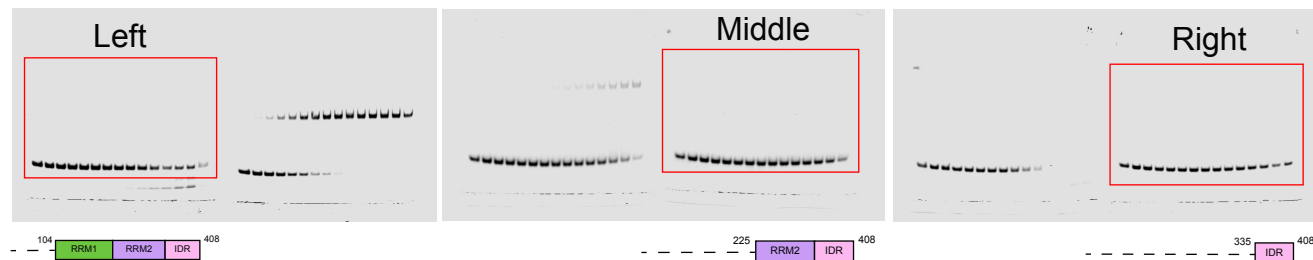

Source Data - Figure 6

Supplement: SourceData F6 — is the source file for Fig. 6. [file jcb_202503087_sourcedataf6.pdf]

Figure 7A

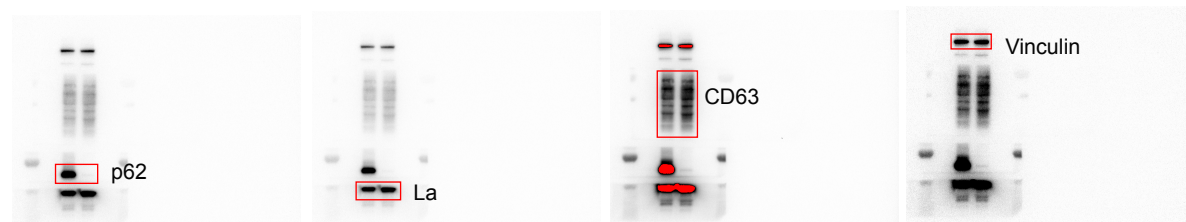

Figure 7B

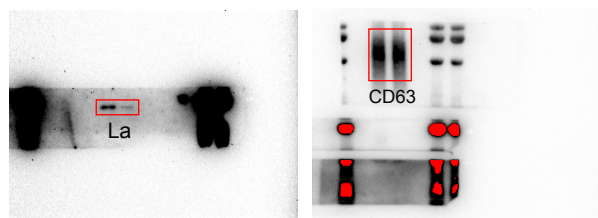

Figure 7D

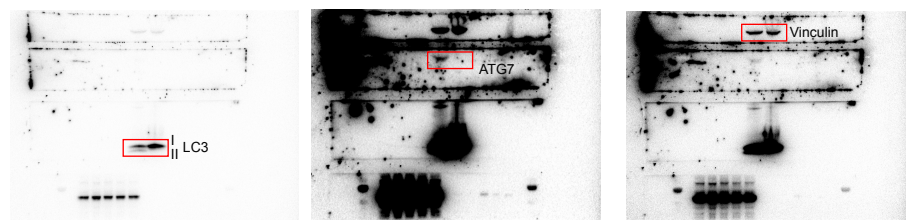

Figure 7E

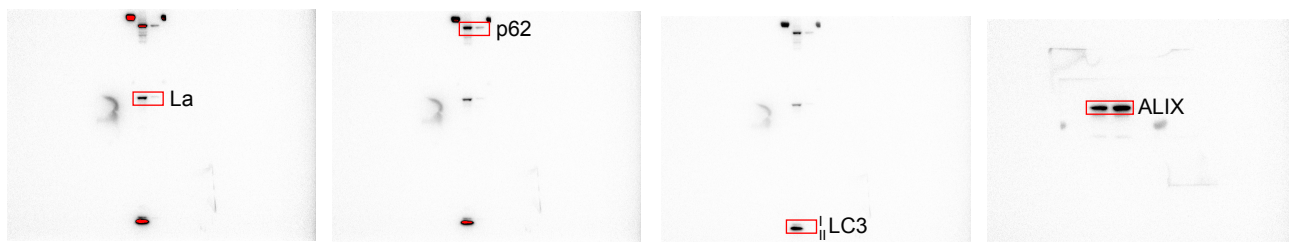

Figure 7G

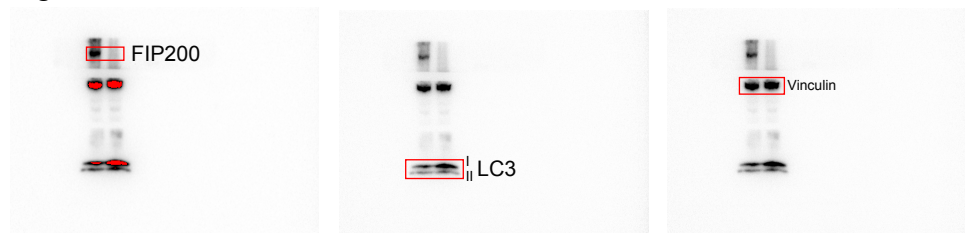

Figure 7H

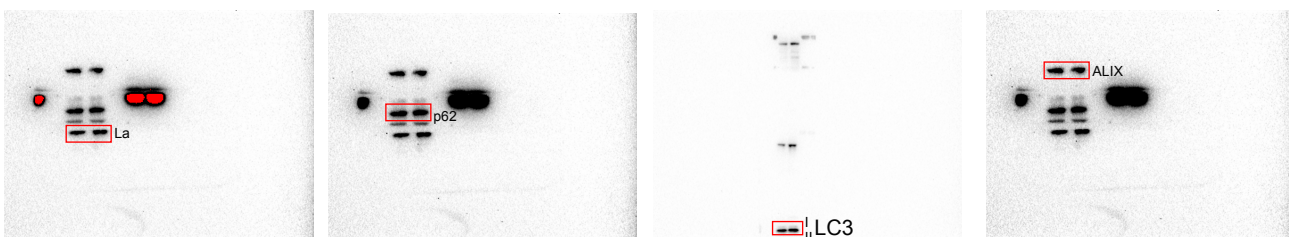

Supplement: SourceData F7 — is the source file for Fig. 7. [file jcb_202503087_sourcedataf7.pdf]

Figure S3A

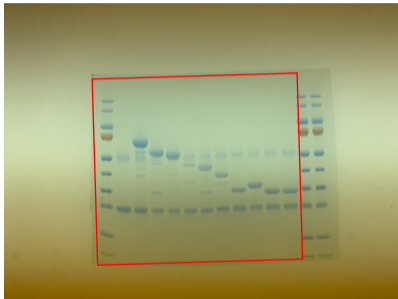

Figure S3B

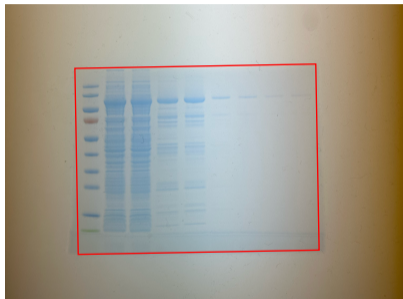

Source Data - Figure S3

Supplement: SourceData FS3 — is the source file for Fig. S3. [file jcb_202503087_sourcedatafs3.pdf]

Figure S4A

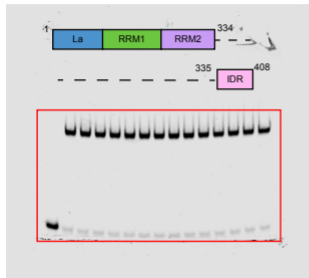

Figure S4B

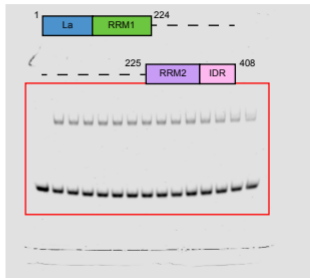

Source Data - Figure S4

Supplement: SourceData FS4 — is the source file for Fig. S4. [file jcb_202503087_sourcedatafs4.pdf]
